# On the Use of Ultrafiltration or Microfiltration Polymeric Spiral-Wound Membranes for Cheesemilk Standardization: Impact on Process Efficiency

**DOI:** 10.3390/foods8060198

**Published:** 2019-06-08

**Authors:** Julien Chamberland, Dany Mercier-Bouchard, Iris Dussault-Chouinard, Scott Benoit, Alain Doyen, Michel Britten, Yves Pouliot

**Affiliations:** 1 STELA Dairy Research Center, Institute of Nutrition and Functional Foods (INAF), Department of Food Sciences, Université Laval, Québec, QC G1V 0A6, Canada; dany.mercier-bouchard.1@ulaval.ca (D.M.-B.); iris.dussault-chouinard.1@ulaval.ca (I.D.-C.); scott.benoit.1@ulaval.ca (S.B.); alain.doyen@fsaa.ulaval.ca (A.D.); yves.pouliot@fsaa.ulaval.ca (Y.P.); 2 Food Research and Development Center (FRDC), Agriculture and Agri-Food Canada, Saint-Hyacinthe, QC J2S 8E3, Canada; michel.britten@canada.ca

**Keywords:** efficiency, microfiltration, ultrafiltration, cheesemilk standardization, process simulation

## Abstract

Ultrafiltration (UF) and microfiltration (MF) are widely-used technologies to standardize the protein content of cheesemilk. Our previous work demonstrated that protein retention of a 0.1-µm MF spiral-wound membrane (SWM) was lower, but close to that of a 10 kDa UF one. Considering that the permeability of MF membranes is expected to be higher than that of UF ones, it was hypothesized that the former could improve the efficiency of the cheesemaking process. Consequently, the objectives of this work were to compare 0.1-µm MF and 10 kDa UF spiral-wound membranes in terms of (1) hydraulic and separation performance, (2) energy consumption and fouling behavior, (3) cheesemaking efficiency of retentates enriched with cream, and (4) economic performance in virtual cheesemaking plants. This study confirmed the benefits of using MF spiral-wound membranes to reduce the specific energy consumption of the filtration process (lower hydraulic resistance and higher membrane permeability) and to enhance the technological performance of the cheesemaking process (higher vat yield, and protein and fat recoveries). However, considering the higher serum protein retention of the UF membrane and the low price of electricity in Canada, the UF scenario remained more profitable. It only becomes more efficient to substitute the 10 kDa UF SWM by the 0.1-μm MF when energy costs are substantially higher.

## 1. Introduction

Dairy products can be manufactured through many processing itineraries, but the most eco-efficient (i.e., having the lowest environmental impact and provides higher incomes) has to be selected to improve the competitiveness of the industry. In the cheese sector, the use of membrane filtration processes to concentrate the milk prior cheesemaking represents one way to improve its profitability and plant capacity [1,2]. Ultrafiltration (UF) is a widely used membrane technology. It allows to concentrate all the milk proteins, notably caseins (CN) and serum proteins (SP), whereas lactose and minerals are collected in the permeate [3,4]. It leads to improved coagulation properties [5,6,7] and increases cheese yield through a higher retention of milk components in the curd [3,6,8].

Microfiltration (MF) is also used for the protein standardization of cheesemilk [3]. As UF membranes, MF ones retain CN in the retentate, but generally have a low SP retention coefficient because of its higher mean pore size [9]. Due to the presence of SP in the MF permeate, it is considered as a “clean whey” since it is free of residues from cheesemaking such as caseinomacropeptite (CMP), cheese fines, colorants, lactic acid, or starter cultures found in traditional whey [10]. Furthermore, SP are found in the permeate in their native form, allowing the production of highly valuable whey protein isolates (WPI) that can be sold for their technological (foaming and gelling) and nutritional properties [11,12].

In a previous study, the performance of both 0.1- and 0.2-µm microfiltration (MF) spiral-wound membranes (SWM) was compared in the context of cheesemilk standardization [13]. Mercier-Bouchard et al. [13] confirmed the low SP retention property of a 0.2-µm MF SWM, but observed that SP are highly retained with a 0.1-µm polymeric MF SWM. A similar conclusion was reported in the literature, revealing the higher SP retention with MF SWM than with ceramic uniform transmembrane pressure (UTP), ceramic graded permeability (GP) membranes or hollow fiber ones [14,15]. In fact, SWM MF membranes, notably the 0.1-µm pore size, suffer from much more severe deposit formation so that the retained casein micelle layer exerts the retention effect.

Considering the largest pore size of MF membranes (higher permeability and hydraulic performance) and the fact that a more valuable permeate could be obtained (even if diluted), it was hypothesized that the cheesemilk standardization could be more efficient with the use of a 0.1-µm polymeric MF SWM, exhibiting a UF-like behavior, instead of a traditional 10 kDa UF SWM. The filtration performance of both membrane types was evaluated in the total recirculation and concentration modes at the pilot scale with skim milk at 50 °C. The cheesemaking efficiency of retentates collected during both filtration methods was also evaluated and compared with that of unconcentrated milk, and an economic assessment of the three scenarios tested was finally presented.

## 2. Materials and Methods

### 2.1. Raw Material

Pasteurized skim milk was purchased from a local dairy farm and stored at 4 °C until MF and UF experiments. These were performed in triplicate, in the recirculation mode, with the same batch of milk divided into three equal volumes of 300 L. For single-stage concentration and diafiltration (DF) modes, a different batch of milk was used for each membrane tested (0.1-μm MF and 10 kDa UF).

### 2.2. Filtration System

A pilot system described previously by Mercier-Bouchard et al. [13] was used for all the filtration experiments (model 393, Tetra Pak Filtration Systems, Champlin, MN, USA). Only one stage was used during this work with two MF or two UF membranes installed in series in the same loop. The 0.1-μm MF SWM was made of polyvinylidene fluoride (PVDF) (membrane v0.1, element specification model 3838, Synder Filtration, Vacaville, CA, USA), and the 10 kDa UF membrane was made of polyethersulfone (PES) (model DS-UH-3838, Microdyn-Nadir, Raleigh, NC, USA). They were mounted horizontally with respective surface areas of 13.38 m^2^ and 10.14 m^2^.

### 2.3. Operational Modes

#### 2.3.1. Total Recirculation Mode

Filtration was performed at 50 °C in the total recirculation mode in order to determine the optimal transmembrane pressure (TMP) to be used in the concentration one. Experiments with 0.1-μm MF membranes were carried out at a TMP of 89.6, 106.9 and 124.1 kPa, as described in Mercier-Bouchard et al. [13], whereas values of 310.4, 379.4 and 447.5 kPa were applied on 10 kDa UF ones. Since neither type was operated with the same TMP, the permeability was chosen to compare them, as described by Methot-Hains et al. [16].

#### 2.3.2. Concentration/DF of Skim Milk

Single-stage batch concentration and discontinuous DF with 1.5 diavolume (DV) were both performed at 50 °C at the optimal TMP, as determined in the recirculation mode, until reaching a targeted mass concentration factor (MCF) of 2.5×. Two DF were carried out (DF #1 and DF #2). Again, the permeability was used to compare the two membrane types. The true protein (TP) rejection coefficient was also calculated [17].

### 2.4. Membrane Fouling Characterization

The resistance-in-series model was applied to evaluate membrane fouling. The membrane resistance (R_m_), reversible resistance (R_rev_), irreversible resistance (R_irrev_), and total resistance (R_tot_) were calculated [4].

### 2.5. Chemical Analysis

Skim milk, retentate, permeate, and whey samples were analyzed according to the methodology described by Tremblay-Marchand et al. [17]. Briefly, the contents of the TP, CN, and non-protein nitrogen (NPN) were determined by the Kjeldahl digestion (AOAC International 991.20, 998.05, and 991.21, respectively). The total solids (TS) and fat (TF) were determined by the forced-air oven drying method (AOAC International 990.20) and the Mojonnier extraction one (AOAC International 989.05), respectively.

### 2.6. Energy Consumption

The electric energy consumption (Wh) of the MF and UF processes were obtained following the calculation of the power requirement (P, W) to operate the filtration system (Equation (1)).

(1) $$P=\sqrt{3}\times U\times I\times \cos\left(\varphi \right)$$

where U, I and cos(*φ*) represent the voltage, current and power factor (0.65), respectively.

The current used to calculate P was that measured at the end of each concentration or diafiltration steps for the feed and recirculation pumps. The specific energy consumption (SEC, Wh per kilogram of permeate that is removed) was determined as follows (Equation (2)):

SEC = (P × Δt)/V_p_ (2)

where Δt and V_P_ represent the time needed to complete concentration and diafiltration steps, and the volume of permeate that is collected during them, respectively.

As in Mercier-Bouchard et al. [13], only electricity used to pump the fluids in the filtration system was considered.

### 2.7. Cheesemaking

The contents of TP of retentates during MF and UF were standardized to a final concentration of 7% (*w/w*) by reincorporating UF permeate. Fat standardization of retentates collected during both filtration types and of unconcentrated milk, was carried out by the addition of unpasteurized cream, obtained from a local dairy farm, in order to reach a final true protein to fat (TP/TF) ratio of 0.65. This low TP/TF corresponds to a high-fat cheesemilk [18]. Following standardization, the retentates were pasteurized at 68 °C for 30 min in a double-jacketed vessel mixer (model UMC-5, Stephan Machinery™, Hameln, Germany). The pH of the cheesemilks was adjusted with glucono-δ-lactone (GDL) to 6.50, and the model curds were produced according to the method described by Lauzin et al. [19] with the following modifications. At the end of the cheesemaking process, the curd was drained in two steps: in cotton cheesecloth over 30 min, and finally by centrifugation at 10,816× *g* for 30 min. The curds were vacuum-packaged and stored at 4 °C until chemical analysis. All experiments were performed in triplicate.

### 2.8. Cheesemaking Efficiency

The cheesemaking efficiency was evaluated with manufacturing yield (Y), moisture-adjusted yield (Y_MA_), and protein and fat recovery (Y_P_ and Y_F_, respectively). These variables were calculated based on the mass of the standardized cheesemilk (Vat) or one of the inputs (Inp). The Y (Equation (3)) and Y_MA_ (Equation (4)) were determined as follows, according to Guinee et al. [1]:

Y_Vat_ (%) = 100 × m_curd_ (kg)/m_Vat_ (kg) (3)

where m_curd_ and m_Vat_ represent the mass of the curd and that of the standardized cheesemilk, respectively.

Y_MAVat_ (%) = Y × (100 − M_curd_)/(100 − M_ref_) (4)

where M_curd_ and M_ref_ represent the moisture (%) of the curd and that of a reference cheese, respectively. The latter corresponded to the cheese made from unconcentrated milk.

The protein recovery in the standardized cheesemilk (Y_PVat_) (Equation (5)) was determined as follows:

Y_PVat_ (%) = 100 × (1 − (m_Pw_/m_PVat_)) (5)

where m_Pw_ and m_PVat_ represent the protein mass in the whey and that in the standardized cheesemilk.

The fat recovery in the standardized cheesemilk (Y_F_) (Equation (6)) was determined as follows:

Y_FVat_ (%) = 1 − (m_Fw_/m_FVat_) (6)

where m_FW_ and m_FVat_ represent the fat mass in the whey and that in the standardized cheesemilk.

### 2.9. Economic Assessment

A process simulation was finally performed to compare the economic assessment of cheesemaking involving the three technological approaches presented previously: from standardized cheesemilk with MF or UF retentates, as well as from unconcentrated milk. It was done using the same parameters (i.e., membrane types, MCF, TP/TF ratio of the cheesemilk, number of DV or DF) as the experimental part, with the following modifications. Regarding the MF and UF scenarios, instead of concentrating the total volume of milk to a MCF of 2.5×, and to dilute it with the permeate, only a fraction was skimmed and concentrated. The retentate obtained was combined with cream and whole milk to standardize cheesemilk to a TP of 5.87% (*w/w*) and a TF of 9.03% (*w/w*). Missing data were interpolated from the ones obtained in the experimental part (i.e., membrane permeation fluxes at specific MCF). It was assumed that filtrations in virtual plants were performed in three-stage filtration systems.

The simulation considered a 1,000,000 kg daily delivery of whole raw milk (TP = 3.27% (*w/w*), TF = 3.97% (*w/w*), lactose = 4.81% (*w/w*), TS = 12.8% (*w/w*) [20]), cream (TP = 0.62% (*w/w*), and TF = 45.00% (*w/w*)) in virtual plants operating 265 days per year. Neither the cleaning or sanitation steps, nor the human resource requirements were taken into account. The simulation focused on the filtration and cheesemaking processes.

### 2.10. Statistical Analysis

Significant differences of each variable were detected by a one-way analysis of variance (one-way ANOVA) with RStudio software (v.1.1.463, [21]) using the Agricolae package (v.1.3-0, [22]). They were evaluated by the Fisher’s least significant difference (LSD) test (*p* < 0.05).

## 3. Results

### 3.1. Effect of TMP on Normalized Permeation Flux in the Total Recirculation Mode

**Figure 1:**
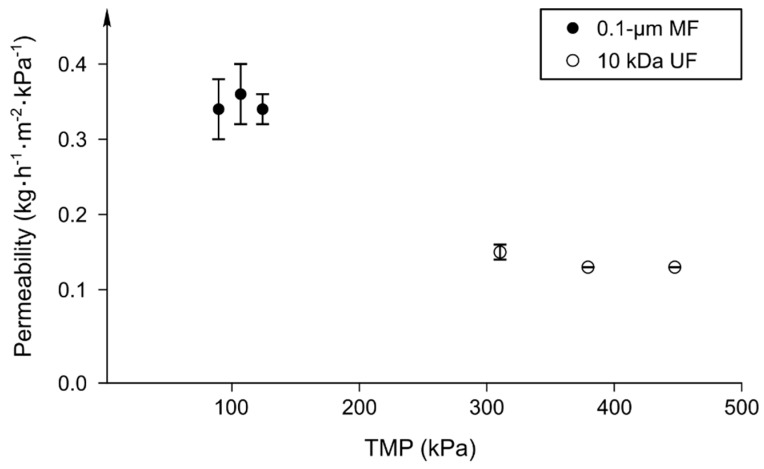
Permeability at different transmembrane pressures (TMP) during microfiltration (MF) and ultrafiltration (UF) of pasteurized skim milk (T = 50 °C) with 0.1-μm MF (●) and 10 kDa UF (○) membranes, respectively (*n* = 3, ± standard deviation [SD]).

Three TMP were tested in the total recirculation mode for MF and UF of skim milk at 50 °C. As shown in Figure 1, no limiting flux was reached between 89.6 kPa and 124.1 kPa for the MF type, and between 310.4 kPa and 447.5 kPa for the UF one. Consequently, the highest TMP obtained for both membranes was selected for further concentration and diafiltration experiments. At these TMP, the permeation flux normalized per unit of pressure was of 0.34 and 0.13 kg h^−1^ m^−2^ kPa^−1^ for the MF and the UF membranes, respectively (Figure 1).

### 3.2. Effect of Concentration and Diafiltration Modes on Normalized Permeation Flux

**Figure 2:**
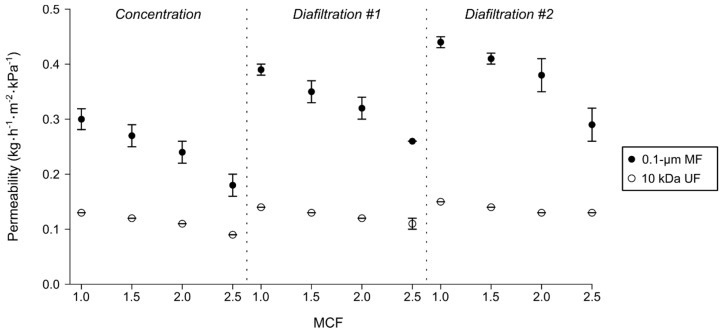
Normalized permeate flux as a function of the mass concentration factor (MCF) with 0.1-μm MF and 10 kDa UF membranes during concentration and two subsequent diafiltration steps (DF) of pasteurized skim milk at 50 °C (*n* = 3, ±SD).

During concentration and DF of skim milk, the difference between the permeability of the membrane types varied significantly at all the MCF tested (*p* < 0.05) (Figure 2). The DF performed with both membranes increased the initial permeability of each reconcentration step. For example, the value for the MF type was of 0.39 kg h^−1^ m^−2^ kPa^−1^ and 0.44 kg h^−1^ m^−2^ kPa^−1^ at the beginning of the first and second DF, respectively, while the initial one was of 0.30 kg h^−1^ m^−2^ kPa^−1^ (Figure 2). The permeability decreased with the MCF during the concentration and both DF steps with the MF and UF membranes, but the flux reduction rate was similar in all the processing steps (Figure 2). It was, however, greater during MF with values between 34.4% (DF #2) and 38.2% (concentration), whereas those obtained during UF were between 13.4% (DF #2) and 31.5% (concentration) (Figure 2).

### 3.3. Effect of Concentration and Diafiltration Modes on Retentate Composition

Globally, the UF membrane had a higher TP rejection coefficient (*p* < 0.05) (Table 1). Consequently, the TP content of permeates collected during MF was always significantly higher than the those during UF (*p* < 0.05), but no significant difference was found regarding the TP of 2.5× UF and MF retentates after the second DF (*p* > 0.05) (Table 1). The transmission of TP in the permeate during MF corresponded to around 11% of the TP of the skim milk. The DF effectively reduced the TS content of 2.5× retentates (MF and UF). For example, the TS content of the retentate during MF was reduced from 14.97 ± 0.65% (*w/w*) after the first concentration step to 11.48 ± 0.42% and 9.88 ± 0.02% (*w/w*) following the first and the second DF, respectively (*p* < 0.05) (Table 1). After the second DF, the 2.5× retentates (UF and MF) had the same TS content (10.09 ± 1.77% and 9.88 ± 0.02% (*w/w*), respectively) (*p* > 0.05) (Table 1). Even if the retentates were diafiltered, their contents of NPN did not differ significantly at the end of each concentration and DF steps (*p* > 0.05) (Table 1). The NPN concentrations were possibly too low to observe differences.

### 3.4. Effect of Concentration and Diafiltration Modes on Energy Consumption

UF was more energy-demanding than MF, during the concentration or both diafiltration steps (*p* < 0.05) (Table 2). Considering the whole process, UF had a SEC of 13.31 ± 0.23 Wh per kilogram of permeate that is removed, whereas that of MF was of 8.59 ± 0.50 Wh per kilogram of permeate removed (Table 2). During MF, the concentration step was more energy-demanding than both diafiltration steps (*p* < 0.05), but the electricity used for these was similar (*p* > 0.05) (Table 2). During UF, the electricity consumption of each step was significantly different (*p* > 0.05) (Table 2).

### 3.5. Effect of Concentration and Diafiltration Modes on Membrane Fouling

The MF and UF membranes had a similar R_rev_ (*p* > 0.05) (Table 3). However, the latter had a higher R_m_, R_irrev_ and R_tot_ than the former (*p* < 0.05) (Table 3). The R_tot_ of the UF membrane type was 2.25 times higher than that of the MF one (Table 3). The R_irrev_ of the UF membranes represented 42% of its R_tot_, whereas that of the MF membranes represented 26% of its R_tot_. Furthermore, the fouling type of the latter was rather reversible than irreversible. Its R_rev_ was 2.31 times higher than its R_irrev_, while it was only 1.13 times higher with the UF membrane (Table 3).

### 3.6. Cheesemaking Efficiency of Unconcentrated Cheesemilk, and Standardized with MF and UF Retentates

The higher moisture-adjusted yield (Y_MAVat_) was obtained with the MF retentate (29.67 ± 0.51%, *p* < 0.05), but the use of UF also permitted to increase Y_MAVat_ compared to unconcentrated cheesemilk (*p* < 0.05) (Table 4). The MF retentate also allowed higher cheesemilk protein (Y_PVat_) and fat (Y_FVat_) recoveries in cheese (90.73 ± 0.13% and 96.91 ± 0.26%, respectively), which also significantly increased the FDM ratio (*p* < 0.05) (Table 4). The use of the same TP/TF ratio in the cheesemilk in the three scenarios allowed a similar draining behavior, revealed by a similar MNFS ratio (67.69 to 67.85) between the three cheeses (*p* > 0.05) (Table 4).

### 3.7. Economic Assessment of MF and UF Approaches through a Process Simulation

The results obtained in the experimental part were used to perform a process simulation in virtual plants receiving 1,000,000 kg of whole raw milk daily (Table 5 and Table 6). Similar patterns between the Y_MA_ observed in the experimental part and the predicted Y_Vat_ (calculated with cheeses having the same moisture content) presented in Table 5 were observed. It is, however, important to mention that the Y_Vat_ predicted in the process simulation was lower (i.e., by 1.16% for the UF approach); the difference between Y_Vat_ of MF and UF scenarios was different as well.

The process simulation confirmed the interest of using filtration processes with the TP/TF ratio of the cheesemilk used in this study, to increase the manufacturing yields calculated from the mass of the inputs (Table 5), and to reduce the operating costs (Table 6). As a fraction of SP are transmitted in the MF permeate, a lower protein recovery from the inputs (Y_PInp_) was observed in the MF scenario compared to the UF one (Table 5). It remained, however, higher than with the unconcentrated milk.

## 4. Discussion

Even though previous works confirmed the high SP retention coefficient value of MF SWM [13,23], notably in comparison with ceramic ones [14], that of the 10 kDa UF SWM was significantly higher in this study (Table 1). The MF membrane had the advantage of a lower hydraulic resistance (Table 3) and a higher permeability at every MCF tested (Figure 2), which was in accordance with its SEC that was low (Wh per kilogram of permeate collected) compared to the UF one (Table 2). As seen in the process simulation, the cheesemilk standardization through MF would require a larger membrane surface area, due to a lower permeation flux at its optimal TMP (despite a higher permeability), but would be less energy-demanding (Table 6).

In the cheesemaking facility, the fat-enriched MF retentate exhibited a higher efficiency in terms of vat yields, as well as protein and fat recoveries (Table 4). Indeed, UF- and MF-standardized milk had the same TP/TF ratio, but the latter retentate had a slightly higher CN/SP one, which possibly contributed to enhancing milk coagulation properties and increased its constituent recoveries [24,25,26]. Conversely, the standardized cheesemilk made from unconcentrated milk had the poorer performance. The same TP/TF should have led to a similar fat loss to other scenarios, but its lower TP content possibly led to a weaker structure of the protein matrix, which is thus conductive to a lower retention of fat (Y_FVat_) [1]. The fat-enriched UF retentate showed a lower protein retention from the standardized cheesemilk (Y_PVat_) compared to the MF one (Table 4), due to the higher content of SP lost in the whey. However, the UF-standardized milk exhibited a higher protein retention than the unconcentrated milk, because a lower volume of whey was generated, and with that a lower loss of SP, as confirmed previously for the manufacture of Cheddar cheese [1,27]. In other studies [1,28,29], the enrichment in proteins with MF or UF retentates did not increase their recovery from input material (milk, cream and dairy ingredients) to cheeses. However, the cheeses that were made during the present studies had a higher moisture content (lower volume of whey expelled) and a lower TP/TF ratio that was high, which possibly affected both protein and fat recoveries.

Overall, both scenarios involving cheesemilk standardization with dairy retentates had a lower operating cost per kilogram of cheese and would increase the margin of the cheesemaking process in comparison with the unconcentrated scenario (Table 6), as also predicted by Papadatos et al. [30]. However, three elements may have biased results: (1) the cheesemilks had a high fat content, which generated important cream expenditures; (2) they also had a different CN content, due to the use of a TP/TF ratio; and (3) the cheesemaking performance of the MF scenario was evaluated directly on the retentate instead of the milk standardized with the latter, as generally performed in the industry [28]. The first two elements appeared more important, as the UF scenario remained the more efficient even with a possible overestimation of the MF cheesemilk performance. Indeed, the 2.5× UF retentate had a higher TP content than the MF one due to the transmission of SP in the MF permeate. Consequently, a higher volume of cream input (6485 kg) was needed in the UF scenario. It increased the operating cost of the UF process by 26,996 Can$ (Table 6). In fact, no bias would occur with the use of a CN/TF ratio, since MF and UF have a similar CN content. This statement was confirmed by an uncertainty analysis performed with a CN/TF instead of a TP/TF ratio of 0.65 (Table 7). With the CN-based standardization target, only slight differences were observed between MF and UF scenarios in terms of inputs or filtration expenditures. The UF scenario was however the more efficient with an operating cost (per kilogram of cheese) 0.5% lower than the MF one, and with a higher Y_Inp_ (Table 7), again due to its higher SP retention. In countries where the energy price is higher, the conclusion could be different. In fact, with the Canadian milk price, the MF scenario would become the more profitable if the one for electricity was 6.4 times higher.

## 5. Conclusions

Contrarily to the previous hypothesis proposed by Mercier-Bouchard et al. [13], even if the MF scenario was the less energy-demanding, notably because of the higher permeability of MF membranes, and had better cheesemaking performance of the MF retentate (calculated from the cheesemilk data collected), the UF one remained more efficient. In fact, with the low cost of electricity in the Canadian economic context, the gain associated with the lower use of energy with the 0.1-µm MF SWM was not sufficient to compensate the loss of SP in the MF permeate, as low as it was. By focusing only on the milk standardization and cheesemaking steps, the 0.1-µm MF SWM could not substitute the 10 kDa UF one without a substantial increase in energy costs (i.e., with energy costs at least 6.4 times higher). A further study considering the processing costs of cheese whey, MF and UF permeates as well as diafiltrates will be necessary to draw definitive conclusions on the economic assessments. It should help to determine if processing diluted MF permeates generated from 0.1-µm MF SWM could contribute to increase the profitability of the MF process over the UF one.

## Figures and Tables

**Table 1:** Mean composition of skim milk retentates and permeates during MF and UF.

Sample	Membrane Type	Filtration Step	MCF	TP (% *w/w*)	NPN (%*w/w*)	TP Rejection Coefficient	Total Solids (%*w/w*)
Retentate	0.1-µm (MF)	Concentration	1.0×	3.13 ± 0.12 ^cd^	0.15 ± 0.01 ^a^	97.07 ± 0.75 ^d^	8.77 ± 0.15 ^de^
			2.5×	7.08 ± 1.62 ^b^	0.09 ± 0.03 ^abcd^		14.97 ± 0.65 ^a^
		DF #1	1.0×	3.05 ± 0.22 ^cd^	0.07 ± 0.01 ^bcd^	97.11 ± 0.52 ^d^	5.25 ± 0.17 ^g^
			2.5×	8.94 ± 0.45 ^a^	0.13 ± 0.06 ^abc^		11.48 ± 0.42 ^b^
		DF #2	1.0×	2.92 ± 0.11 ^d^	0.05 ± 0.01 ^d^	98.92 ± 0.21 ^bc^	4.56 ± 0.87 ^g^
			2.5×	8.35 ± 0.08 ^ab^	0.14 ±0.11 ^ab^		9.88 ± 0.02 ^cd^
	10 kDa (UF)	Concentration	1.0×	3.53 ± 0.56 ^cd^	0.13 ± 0.02 ^abc^	99.36 ± 0.06 ^ab^	8.48 ± 0.11 ^ef^
			2.5×	8.91 ± 0.82 ^a^	0.14 ± 0.02 ^ab^		16.12 ± 0.05 ^a^
		DF #1	1.0×	4.40 ± 1.22 ^c^	0.07 ± 0.01 ^bcd^	98.38 ± 0.13 ^ab^	7.25 ± 1.01 ^f^
			2.5×	9.23 ± 0.27 ^a^	0.09 ± 0.03 ^abcd^		11.96 ± 0.74 ^b^
		DF #2	1.0×	3.67 ± 0.48 ^cd^	0.06 ± 0.04 ^cd^	99.82 ± 0.04 ^a^	4.92 ± 0.57 ^g^
			2.5×	9.15 ± 1.50 ^a^	0.11 ± 0.07 ^abcd^		10.09 ± 1.77 ^c^
Permeate	0.1-µm (MF)	Concentration	2.5×	0.22 ± 0.11 ^a^	0.09 ± 0.06 ^ab^	-	5.93 ± 0.22 ^a^
		DF #1	2.5×	0.26 ± 0.04 ^a^	0.15 ± 0.08 ^a^	-	2.06 ± 0.03 ^b^
		DF #2	2.5×	0.09 ± 0.02 ^b^	0.06 ± 0.02 ^bc^	-	0.76 ± 0.01 ^c^
	10 kDa (UF)	Concentration	2.5×	N.D.	0.15 ± 0.01 ^a^	-	5.50 ± 0.06 ^a^
		DF #1	2.5×	N.D.	0.05 ± 0.01 ^bc^	-	1.41 ± 0.77 ^bc^
		DF #2	2.5×	N.D.	0.02 ± 0.00 ^c^	-	0.92 ± 0.56 ^c^

^a–h^ Values in the same column with a common superscript are not significantly different (LSD least significant difference test, *p* > 0.05, *n* = 3, ±SD). Retentates and permeates were compared separately. TP: true protein (TN−NPN); NPN: non-protein nitrogen × 6.38; TN: total nitrogen × 6.38; DF: diafiltration, N.D.: not detected.

**Table 2:** Energy consumption of feed and recirculation pumps during MF ^1^ and UF ^1^ of skim milk at 50 °C.

Filtration Step	Specific Energy Consumption ^1^ (Wh per kg of Permeate Removed)	
	MF	UF
Concentration	10.81 ± 0.77 ^bA^	15.60 ± 0.28 ^aA^
Diafiltration #1	8.05 ± 0.40 ^bB^	12.89 ± 0.33 ^aB^
Diafiltration #2	6.92 ± 0.51 ^bB^	11.47 ± 0.10 ^aC^
Whole process	8.59 ± 0.50 ^b^	13.31 ± 0.23 ^a^

^1^ The optimal transmembrane pressure (TMP) was used to perform MF (124.1 kPa) and UF (448.2 kPa) until reaching a mass concentration factor (MCF) of 2.5× during the concentration and diafiltration steps (*n* = 3, ± SD). ^a,b^ Values in the same row with a tiny common superscript letter are not significantly different (one-way ANOVA, *p* > 0.05, *n* = 3). ^A–C^ Values in the same column with a common capital superscript letter are not significantly different (LSD least significant difference test, *p* > 0.05, *n* = 3).

**Table 3:** Hydraulic resistance of MF and UF membranes following concentration and diafiltration processes.

Membrane Type	Resistance Type (10^−13^ m^−1^)			
	R_m_	R_rev_	R_irrev_	R_tot_
MF (0.1-μm)	0.47 ± 0.01 ^b^	1.87 ± 0.92 ^a^	0.81 ± 0.13 ^b^	3.15 ± 0.96 ^b^
UF (10 kDa)	0.72 ± 0.05 ^a^	3.38 ± 0.30 ^a^	3.00 ± 0.39 ^a^	7.09 ± 0.23 ^a^

^a–b^ Means in the same column with a common superscript are not significantly different (*p* > 0.05). R_m_: membrane resistance, R_rev_: reversible resistance, R_irrev_: irreversible resistance, R_tot_: total resistance.

**Table 4:** Cheesemaking efficiency of MF and UF retentates obtained with spiral-wound membranes.

Indicator	Unconcentrated Cheesemilk	0.1-μm MF Retentate	10 kDa UF Retentate
TS (%)	51.11 ± 0.23 ^b^	53.30 ± 1.29 ^a^	52.09 ± 0.50 ^ab^
MNFS (%)	67.69 ± 0.61 ^a^	67.85 ± 1.53 ^a^	67.75 ± 0.67 ^a^
FDM (%)	54.34 ± 0.92 ^c^	58.50 ± 0.93 ^a^	56.23 ± 0.87 ^b^
Y_Vat_ (%)	14.13 ± 0.25 ^b^	28.46 ± 0.53 ^a^	28.21 ± 0.47 ^a^
Y_MAVat_ (%)	14.13 ± 0.30 ^c^	29.67 ± 0.51 ^a^	28.75 ± 0.42 ^b^
Y_FVat_ (%)	89.46 ± 0.41 ^c^	96.91 ± 0.26 ^a^	96.29 ± 0.12 ^b^
Y_PVat_ (%)	76.15 ± 0.26 ^c^	90.73 ± 0.13 ^a^	86.17 ± 0.20 ^b^

^a–c^ Values in the same rows with a common superscript are not significantly different (Fisher’s LSD test, *n* = 3, ±SD, *p* > 0.05). TS: total solids, MNFS: moisture in nonfat substances, FDM: fat content in dry matter, Y_Vat_: mass yield reported on the mass of cheesemilk in vats, Y_MA_: moisture-adjusted yield with the moisture content of the cheese made from unconcentrated milk, Y_FV_: fat recovery from the standardized cheesemilk, Y_PV_: protein recovery from the standardized cheesemilk.

**Table 5:** Mass balance obtained from a process simulation of cheesemaking approaches involving milk concentration or not in virtual plants receiving 1,000,000 kg of whole milk daily.

Indicator	Scenarios		
	Unconcentrated Milk	MF-Standardized Cheesemilk	UF-Standardized Cheesemilk
Inputs (kg)			
Whole milk	1,000,000	1,000,000	1,000,000
Cream	24,070	23,178	30,012
Calcium chloride	143	73	78
Starter culture ^1^	20	10	11
Coagulant enzyme	232	119	127
Outputs (kg)			
Cheese ^2^	145,049	147,531	154,592
Cheese whey	879,995	376,863	405,647
Permeate	-	602,466	628,049
Diafiltrate	-	1,204,932	1,256,099
Predicted mass yield ^3^ (%)			
Y_Vat_	14.16	28.13	27.59
Y_Inp_	14.16	14.42	15.01
Y_PInp_	76.15	84.57	85.73

^1^ Lyophilized culture. ^2^ Cheese had a total solid (TS) content of 51.11%, as cheese made with the unconcentrated milk in the experimental part. Resulting masses were obtained from the protein and fat recoveries measured in the experimental part. Lactose content had the same concentration in the aqueous phase of the cheese as in the cheesemilk. ^3^ The mass yields were identical to the moisture-adjusted ones since cheeses made by the three scenarios had a similar composition. Y_Vat_: mass yield reported on the mass of cheesemilk in vats, Y_Inp_: mass yield reported on the mass of inputs, namely raw milk and cream, Y_PInp_: protein recovery from the inputs.

**Table 6:** Economic assessment calculated from a process simulation of cheesemaking approaches involving milk concentration or not in virtual plants receiving 1,000,000 kg of whole milk daily.

Indicator	Price	Scenarios		
		Unconcentrated Milk	MF-Standardized Cheesemilk	UF-Standardized Cheesemilk
Expenditures				
Inputs				
Whole milk	84.56 Can$ 100 kg^−1^	845,600	845,600	845,600
Cream	3.95 Can$ kg^−1^	95,077	91,553	118,549
Calcium chloride	2.23 Can$ kg^−1^	320	164	175
Starter culture ^1^	600 Can$ kg^−1^	12,289	6293	6723
Coagulant enzyme	28.32 Can$ kg^−1^	6583	3371	3602
Subtotal	Can$	959,869	946,981	974,649
Filtration				
Membrane replacement ^2^	0.23 Can$ m^−2^ day^−1^	0	1,169	714
Electricity	0.0327 Can$ kWh^−1^	0	508	821
Power	0.42 Can$ kW^−1^	0	821	1327
Subtotal	Can$	0	2498	2862
Operating costs	Can$	959,869	949,479	977,511
	Can$ kg of cheese^−1^	6.62	6.44	6.32

^1^ Lyophilized culture. ^2^ Membrane replacement cost considers a replacement once per year. Filtration processes were operated 8 h per day, 265 days per year, in a three-stage system.

**Table 7:** Uncertainty analysis using a CN/TF ratio of 0.65 instead of a TP/TF ratio.

Indicator	Price	Scenarios		
		Unconcentrated Milk	MF-Standardized Cheesemilk	UF-Standardized Cheesemilk
Expenditures				
Inputs	Can$	879,859	891,849	895,681
Filtration	Can$	0	2431	2815
Operating costs	Can$	879,859	894,280	898,496
	Can$ kg of cheese^−1^	6.87	6.55	6.52
Y_Inp_	%	12.85	13.53	13.65
Y_PInp_	%	76.15	85.10	86.17

Y_Inp_: mass yield reported on the mass of inputs, Y_PInp_: protein recovery from the inputs.
